# Supplementary material for: Chromothripsis during telomere crisis is independent of NHEJ, and consistent with a replicative origin
Source: Genome Res. 2019 May;29(5):737–49. doi: 10.1101/gr.240705.118 (PMC6499312; doi:10.1101/gr.240705.118)
Supplement: Supplemental Material [file supp_gr.240705.118_Supplemental_file_1.zip › contigs/annotated_contigs/DB37/contig.2.DB37_length_422_mean_cov_4.89099526066.docx]

**DB37_length_422_mean_cov_4.89099526066**

```
GGTCCACTTTAAATTCAAACAAAGTTGGCTTTCTTT|TTT|TTTTTCAATTTTACAACCTTAGTTTTTTGTATTTTTTTTATTATTACA
 >chr5:56721932-56721968 - E=4e-10 p=6e-03  >chr5:56720395-56720778 - E=1e-219
TTCAAAAAAACAAACAATAAGATGAGGACAGGGTTGGGGCATATAAAACAGGTCCCTAAATAGATGACAGACCTGCATACAGTTACCAT

AGAAGTATACAGTACCCCTCTCCCCTCAGTTAGGCTATGTATTTGTCTTTTCCACTGTTGTTCAGTAAGTTCCAATGGTTCTACCTTGA

AATAGGTAACAGTCTTTGGAAAAAGTCATTCTAGGTTGTACAAAGGCTCTATATATACAGTTTGTCACAAGAAACAAAGCTTCTTTCCA

AAATTTAAAAAAATTAGTGTGTATTTGTTTTGGTATTTTTTTTATTTTTCAATTTTTTGCAAAGCAAT
```
